# Integrated people-centred primary health care in Greece: unravelling Ariadne’s thread

**DOI:** 10.1017/S1463423619000446

**Published:** 2019-07-25

**Authors:** Christos Lionis, Emmanouil K. Symvoulakis, Adelais Markaki, Elena Petelos, Sophia Papadakis, Dimitra Sifaki-Pistolla, Maria Papadakakis, Kyriakos Souliotis, Chariklia Tziraki

**Affiliations:** 1 Department of Social Medicine, Clinic of Social and Family Medicine, Faculty of Medicine, University of Crete, Heraklion, Greece; 2 Faculty of Medicine and Health Sciences, Linköping University, Linköping, Sweden; 3 Department of Family, Community and Health Systems, WHO Collaborating Center for International Nursing, School of Nursing, University of Alabama at Birmingham, Birmingham, AL, USA; 4 Health Services Research, Care and Public Health Research Institute (CAPHRI), Faculty of Health, Medicine and Life Sciences, University of Maastricht, Maastricht, Netherlands; 5 Division of Cardiac Prevention and Rehabilitation, University of Ottawa Heart Institute, Ottawa, ON, Canada; 6 Faculty of Medicine, University of Ottawa, Ottawa, ON, Canada; 7 Department of Social Work, Technological Educational Institute of Crete, Heraklion, Greece; 8 Faculty of Social and Political Sciences, University of Peloponnese, Corinth, Greece; 9 Reseach Institute, Melabev and Hebrew University, Jerusalem, Israel

**Keywords:** Greece, integrated, multidisciplinary, people-centred care, primary care, primary health care

## Abstract

The 40th anniversary of the World Health Organization Alma-Ata Declaration in Astana offered the impetus to discuss the extent to which integrated primary health care (PHC) has been successfully implemented and its impact on research and practice. This paper focuses on the experiences from Greece in implementing primary health care reform and lessons learned from the conduct of evidence-based research. It critically examines what appears to be impeding the effective implementation of integrated PHC in a country affected by the financial and refugee crisis. The key challenges for establishing integrated people-centred primary care include availability of family physicians, information and communication technology, the prevention and management of chronic disease and migrant and refugees’ health. Policy recommendations are formulated to guide the primary health care reform in Greece, while attempting to inform efforts in other countries with similar conditions.

## Introduction

Over the last 15 years, a large body of research has focused on the need for integrated, multidisciplinary, team-based and people-centred care. In May 2016, the *Framework on integrated people-centred health services* (IPCHS), adopted with overwhelming support by the Member States of the World Health Assembly (WHA), provided renewed direction and political commitment (WHO, 2017) towards the role of primary care (PC) as integral to implementing integrated care. As part of the 40th anniversary of the World Health Organization’s (WHO) Alma-Ata Declaration and the recent Astana Declaration, the WHO has called for a return to its basic tenets, reaffirming the key role of primary health care (PHC) (WHO, 2018a). Revisiting and amending the PHC Declaration offers a unique opportunity to reflect on key achievements and to re-examine how integrated, people-centred care can catalyse PHC-driven universal health coverage (UHC). However, a deeper understanding of these fundamental concepts, and how to deploy them at the community, local, national and cross-border levels is still lacking in many countries. Inter-professional training and team building is also largely lagging behind. Furthermore, necessary supporting frameworks in terms of organisational structures, governance and legislation are either not in place or not comprehensive enough to support transition in many Member States. Available evidence from research efforts developed with significant investment from different European funding mechanisms is more often than not overlooked or underutilised by policymakers when making decisions. This is particularly relevant in terms of PHC and in the context of promoting tenets of the Alma-Ata and the current Astana Declaration (WHO, 2018a). These issues are particularly important when discussing evidence-informed policy-making, especially in countries where accessibility disparities and service inequities are not systematically and adequately addressed.

In this paper, we aim to explore why, despite all efforts undertaken and existing evidence, implementation of the Alma-Ata Declaration did not materialise in Greece, and what should be done differently after the Astana Global Conference. We believe Greece exemplifies the socio-economic, political and education drivers of many countries adversely affected by the financial crisis and austerity measures as well as of any country with underdeveloped PHC or lacking synergies in the context of integrated care provision. Moving beyond lessons learned for Greece, we aim to meaningfully discuss experiences to formulate recommendations to move forward so as to successfully meet the Sustainable Development Goal (SDG) 3, and more specifically, target 3.8 (SDG 3.8) [*Achieve universal health coverage (UHC), including financial risk protection, access to quality essential health care services, and access to safe, effective, quality, and affordable essential medicines and vaccines for all*].

More specifically, we report on the status of integrated people-centred PHC in Greece and describe actions taken to date, highlighting lessons learned from evidence-based research. In this context, we critically examine what appears to be barriers to the effective implementation of a comprehensive PHC system in Greece. We conclude with a set of evidence-informed policy recommendations on the basis of evidence from Greece and the international literature, especially from WONCA consensus papers and collaborative studies. Lessons learned and recommendations on how a country with limited capacity and resources could effectively implement and sustain integrated, people-centred care could be of value to other countries facing similar constraints and challenges.

To serve the aim of this paper, we are using the terms PHC and PC. PHC is an approach to health policy and service provision that includes both services delivered to individuals (including patient pathways) and the general population (White, 2015). PC refers to ‘family doctor-type’ services delivered to individuals, whereas some frameworks (White, 2015; Kringos *et al*., 2010a) use PC to assess PHC components. We have also adopted an operational integration model based on the Donabedian approach (Donabedian, 1976), combining the basic PHC principles presented by Starfield (2009) and the Chronic Care Model (Boult *et al*., 2008). The dimensions of PHC as reported in the work of Kringos *et al*. (2010b) was selected as the most fitting for the Greek health care system.

## The current situation in Greece

Greece first introduced a PC reform jointly with Spain and Portugal, as part of a WHO pilot project to assess different approaches through the operationalisation of key concepts of the Alma-Ata Declaration (WHO, 2018b). In the early 2000s, the reform was widely discussed in the context of drafting and enacting legislation, and the necessity for integrated PC was highlighted for the first time (Souliotis and Lionis, 2004). In 2009, a systematic review exploring the role of integrated PHC within the Greek health policy agenda concluded that it was still in its infancy, recommending a major restructuring of the National Health System (NHS) (Lionis *et al*., 2009). In 2015, Tsiachristas *et al*. (2015) revisited the subject to provide an action plan that would bridge local policy and population needs considering experiences gained from other European settings. At that time, a critical debate, involving both politicians and professionals across specialties, was taking place as a result of major changes that were occurring in the country, and following a number of years with multiple measures of austerity implemented in the context of rationing versus rationalising care.

Since 2010, Greece has been severely affected by the economic crisis and austerity with significant direct impact on the state-funded NHS. As a result, the country refocused its reform efforts on PC with two legislative bills. The first one, Law N° 4238/2014, had a clear fiscal direction on the National Primary Health Care Network (PEDY) and the scope of the Greek National Health Service (EOPYY). This reform introduced the integration of PHC structures and outposts with services of the Social Insurance System. Although conceptually on the right path, the reform faced several difficulties during implementation. It is still debated to what extent this is the direct result of serious lack of manpower, pushing citizens to higher utilisation of private services, including those of specialists, in a country with a long-standing tradition of mixed public and private services, and no gate-keeping system or comprehensive PHC system to start with. The second legislative bill, Law N° 4486/2017, was enacted within months of being posted for public is currently in its early phases of implementation. Partly because of the very low number of general practitioners (GPs), the direction is to strengthen the role of the family physician by assigning it to GPs, internists and paediatricians. As gatekeepers to costlier and specialised health care services and interventions, family physicians are the first point of contact, providing comprehensive and continuous care with a focus on disease prevention and health promotion. The law mandated the opening of more than 200 Local Primary Care Units (TOMY) in urban centres, jointly funded by the Greek State budget and the European Social Fund (ESF) under the National Strategic Reference Framework for a period of three years. These PC Units are team-based and comprise family physicians, nurses, health visitors, administrative staff and in some cases a midwife. A media campaign was launched in 2018 to raise public awareness. As of January 2019, just over 100 TOMY units were fully operational. These recent reform efforts have sparked an intense national debate over the lack of sufficient numbers of family physicians, financial incentives to ensure adequate recruitment and the sustainability of the units beyond the three-year period of funding support by the ESF. The latter necessitates further structural changes across levels of care and reallocation of resources to ensure sustainability.

Despite the above challenges, establishing a fully operational and sustainable integrated PHC system in Greece is more relevant than ever before. A recent survey by the Observatory on Health Reforms, a joint initiative of the University of Peloponnese and the Health Policy Institute, revealed that there are serious and persistent barriers, primarily access to physicians and financial concerns, which have changed the public’s attitudes towards GP-led care pathways (Kanavos and Souliotis, 2017). There is evidence that GP-led pathway to care can act not only as a means to improve access to care for all, but also as a vehicle to guarantee fiscal sustainability for the health system as a whole (Kanavos and Souliotis, 2017).

## Lessons learned from Greek observational research studies relevant to integrated multidisciplinary health care services

From 2012 to 2017, the Clinic of Social and Family Medicine at the Faculty of Medicine of the University of Crete (UoC) carried out several regional and national studies on PC services relevant to the implementation of an integrated PHC system in Greece. Specifically, a study exploring the *scope of practice and training needs* of PHC nurses working in rural Health Centres on the island of Crete found that nursing staff operated within a restricted and task-oriented framework (Markaki *et al*., 2006; 2007; 2009). Job satisfaction was low in terms of daily interactions with colleagues and support from work environment. The need for timely recruitment of young, qualified nursing staff choosing a career in PHC explicitly emerged (Markaki *et al*., 2006). Significant training needs were reported by all nursing personnel, mainly in clinical and research/audit skills. Most respondents expressed a strong desire for continuing education, despite a self-reported adequate knowledge level. Investigators recommended a systematic overview of skill deficits, in relation to skill requirements, implemented regularly by NHS authorities to enhance targeted on-the-job training, based on individual- and group-specific needs (Markaki *et al*., 2009).

*The need for multidisciplinary team-work* in managing health problems was also highlighted in research by Papadakaki *et al*. (2013; 2014) who studied physician readiness to manage intimate partner violence (IPV). The PREMIS (Physician Readiness to Manage IPV) survey was translated and validated into Greek. A pre-post control group design was used to examine an intervention for addressing IPV which involved both GPs and residents in general practice in Crete (Papadakaki *et al*., 2011; 2013). The training programme met a high acceptance by both groups with the intervention group of GPs performing better than the control group on ‘perceived preparedness’ and ‘perceived knowledge’ in both the post-intervention and the 12-month follow-up (Papadakaki *et al*., 2012; 2013). The study found no statistically significant improvements or between group differences in terms of the self-reported detection of IPV cases (Papadakaki *et al*., 2013). This is a good example on how a training program could be designed based on a multidisciplinary approach and implemented effectively in low-resource settings.

*Perceptions of PHC practitioners and patients* regarding the quality of services in rural Greece were also evaluated in a study involving 21 PHC units from two regions of Greece, namely, Epirus and Crete (Sbarouni *et al*., 2012). This research generated recommendations for re-engineering the existing, at the time, system, including: (a) introduction of new technologies and emphasis on system interoperability, (b) GP empowerment, leadership reform and (c) mechanisms for evaluating the quality of services. Areas of concern regarding future development and utilisation of private PHC infrastructure and services were highlighted (Sbarouni *et al*., 2012). A second large study explored patients’ expectations and experiences in Greece using data collected as part of the international QUALICOPC study (Lionis *et al*., 2017). Several barriers were identified in terms of waiting time for appointments, GP access to patient medical history, delivery of preventive services and patient involvement in decision-making (Lionis *et al*., 2017). Patients with chronic disease reported a better experience than respondents without a chronic condition and highlighted the disease-focused orientation of health service delivery. Data gathered may be used to address the quality of services through an increased focus on patient-centred interventions, and people-centred approaches as well as on preventive service delivery.

A nationally funded research project carried out by the UoC focused on *operational integration* and had a two-fold aim (1) to assess the level of operational integration within PHC units by utilising standardised mapping and evaluation of both unit-level and patient-level integration and (2) to develop an optimum model of operational integration tailored to the Greek PHC system (Sifaki-Pistolla *et al*., 2017). Existing patient care pathways were mapped and analysed, and recommendations for an optimum model were made. The developed web platform, based on a strong theoretical framework, can serve as a robust integration evaluation tool and contribute towards optimising patient flow across levels of cares, beyond unit level. This work could serve as a first step towards restructuring and improving PHC services within a financially restrained environment (Sifaki-Pistolla *et al*., 2017). A recently published paper by the UoC research team focused on how health care delivery data, collected from capacity building and research initiatives, can facilitate effective planning and integration of public health into PHC (Lionis *et al*., 2018b).

Research activities summarised here focus on the PHC working environment through the lens of physicians, nurses, other health care providers, and most importantly, patients and people across communities. All studies contribute towards enhancing knowledge, skills, cooperation and team work in the direction of building an integrated PHC model with the patient and family as an empowered and integral part of the care team. Thus, ensuring a dynamic relationship and establishing sound ground for shared decision-making are a key issue highlighted in the WHO Astana Declaration.

## Key challenges for establishing integrated people-centred PC in Greece

Based on the study by Kringos *et al*., Greece was classified among the countries with weak PC in terms of access, continuity and comprehensiveness, while it was rated as strong on coordination (Kringos *et al*., 2013). Although, the current NHS legislative framework promotes some of the key principles in the Alma-Ata Declaration, there are several barriers impeding progress. We review here the key barriers and challenges affecting PC services in Greece today.

### Availability of family physicians

Human resources is considered a key factor to improving health care and the following demonstrates its relevance to integrated PC. While Greece has the highest per capita rate of licensed physicians among EU Member States (632 per 100 000 inhabitants in 2015), these are primarily specialists (Economou, 2015). This ratio is considerably higher than any of the other EU Member States; Austria (510) and Portugal (461) had the next highest ratios of physicians licenced to practice and along with Lithuania (434) were the only other Member States to record over 430 physicians per 100 000 inhabitants. In contrast, Greece reports the lowest per capita rate of GPs (0.28 per family practitioner per capita) in Europe (Economou, 2015; Eurostat, 2018). The per capita ratio of registered nurses is also among the lowest in Europe (Economou, 2015). The high density of specialists and disproportionate ratio of specialist to GPs create challenges and barriers to PC reform. Training programs with a standardised curriculum for general practice have only recently been developed, while the academic capacity of this specialty needs more support. Furthermore, the departure of large numbers of physicians during the austerity period (brain drain) has compounded the paucity of GPs. It has been reported that more than 18 000 Greek physicians have left Greece to seek employment and/or better work conditions (Economou, 2015). In October 2013, the Athens Medical Association reported that approximately 7000 doctors of all specialties left between 2008 and 2013 after the onset of the financial crisis (Economou, 2015). This leads to imbalances in service provisions and highlights the direct effect of ‘brain drain’ on efficient health care service delivery (Economou, 2015).

### Information and Communication Technology (ICT)

Greece is not the only European country suffering from lack of interoperable systems – indeed this is a well-recognised issue affecting cross-border health care across countries. Nevertheless, the lack of a uniform electronic health record (EHR) across regions and settings, linked effectively to the centralised prescribing and referral services, has been a significant impediment to progress. Other barriers to achieving integrated PC include (a) lack of a national electronic health monitoring platform to support communication and patient registries, (b) episodic health screening, (c) fragmented coordination of referrals for chronic disease management, in particular cancer care, (d) weak social services support, and (e) increased demand for services due to migration and refugee health problems. Collaborative projects on integrated care services have shown that delays in the transfer of ITC linkage and the socio-economic environment may contribute to negative results when advanced training initiatives are launched in different settings (Barberan-Garcia *et al*., 2014; Hernandez *et al*., 2015). Although Greece has adopted and applied medical informatics education, including multidisciplinary postgraduate courses and large participation in EU-programs informatics research, with palpable scientific contributions, these remain fragmented efforts. Although local experiences are reported in the literature (Kounalakis *et al*., 2003; Samoutis *et al*., 2007), the country still struggles to implement a uniform and universal EHR and interoperable e-health applications. According to research findings, major implementation barriers are physicians’ beliefs that EHR adoption affects workflow, physicians’ ethical concerns, lack of incentives, system breakdowns, software design deficiencies, transition difficulties and lack of familiarity with electronic devices (Samoutis *et al*., 2007). Across Europe, countries that have invested in workforce and ICT infrastructure, understanding how cultural differences and capacity affect teamwork, have succeeded in reaching with health system improvement goals (Auffray *et al*., 2016).

### Prevention and management of chronic disease

Within the EU of 28 member countries, Greece reports among the highest rates of chronic diseases (lung cancer, lung diseases and CVD) and very high rates of CVD risk factors, including the highest rate of tobacco use and growing rates of obesity, metabolic syndrome and cancer. Preventing exposure to NCD-causing risks, such as physical inactivity, tobacco smoking, air pollution and other similar risks can be tackled through a toolkit of tested and affordable policy interventions. The ‘natural’ setting for utilising such a toolkit and implementing interventions is found at the PC level through PHC provision. PHC must work synergistically with strong public health policy and strategies ranging from strong enforcement of policies on smoke-free public places to increased taxation of unhealthy products.

The primarily acute care-focused Greek NHS accounts for the very low rates of preventative health screening and vaccination relative to other EU countries. This results in delayed diagnosis and treatment of risk factors and disease. Strong working relationships between public health and PHC to support health screenings and pre-symptomatic care delivery are critical for addressing the burden of NCD nationally. Strengthening ICT system supports also plays a critical role in NCD management. For example, monitoring practice performance, phone and email contacts, development of communication and cultural interaction skills have all been found to improve rates of colorectal cancer screening delivery, offering a good example for implementing an integration care model (Triantafillidis *et al*., 2017). Considering the low levels of systematic screening program deployment in Greece, we can presume that curative services have been disproportionally affected within NHS priorities, in comparison to preventive services. Service fragmentation with functional disruption between primary and secondary care may be attributed to the lack of any systematic effort of health integration for a long period of time. Examining the example of cardio-metabolic disease (CMD), a recently published European systematic review revealed that younger age, smoking, low education and attitudes, such as being or not being worried about the outcome, low perceived severity or susceptibility and negative attitude towards health checks or prevention were barriers for participation in cardiovascular health checks (deWaard *et al*., 2018a). In a subsequent European study, despite the fact that most GPs considered selective CMD prevention as useful, it was not universally implemented. The biggest challenge was inviting individuals for risk assessment, while the involvement of stakeholders was identified as important (deWaard *et al*., 2018b).

Greece is still far from integrating self-management and self-care in chronic disease management. Investing in the development of expanded information and emotional support networks, and attendance of community organisations in decision-making processes have been shown to result in better self-management capabilities (Koetsenruijter *et al*., 2016). Strengthening health system understanding and investment in known self-management strategies and interventions will be required to compliment actions in the broader NHS.

### Migrant and refugee health

Another challenge for the current PHC system in Greece has been introduced as a direct result of the migrant and refugee crisis in Europe. In many European countries, providing newly arrived migrants and refugees a more systematic health-reception, based on a holistic approach by a multidisciplinary team, will not only benefit migrants and refugees but also will protect the public health of host countries (Leder *et al*., 2017). As part of EUR-HUMAN project, UoC explored the barriers and facilitators for an accessible, acceptable and appropriate – culturally and linguistically relevant PHC (Lionis *et al*., 2018a; vanLoenen *et al*., 2018). This approach was informed and tested in five European countries with diverse health care systems. The inclusion of care recipients in early dialogue for needs, assessments and to elicit their wishes and preferences, results in higher engagement of both professionals and migrants/refugees and empowerment to take more proactive roles. Lessons learned included revisiting the feasibility assessment, and the toolkit generated could readily be used across settings to establish positive initial interaction and smoother conditions for the start of implementation journeys. Many shortcomings became evident, such as the absence of clear policies on entitlement to care, while legal restrictions on health care access for marginalised migrants resulted in numerous cases of ambiguous paths, decisions and delays in the provision of appropriate care. In particular, for irregular migrants that may remain undocumented until they reach the care system with an acute episode or for those having been refused asylum, the structural barriers were seriously impeding access and appropriate care delivery (Lionis *et al*., 2016; Teunissen *et al*., 2016; Papadakaki *et al*., 2017).

It is important to remember that UHC cannot be achieved in its true essence without providing for refugee and migrant groups. It is also important to consider an appropriate PHC versus other acute forms of care for these groups. Support at the primary level can facilitate not only smoother integration including entry into the health care system, but also appropriate surveillance and monitoring in the interest of public health, without compromising the rights of people. While the current economic conditions determine key aspects of Greek PC delivery, several practical and training limitations (e.g., number of interpreters, access to location, medical interpreting requires lengthy and specialised training) have surfaced. Interestingly, there is no single European coordinating mechanism to provide guidelines, high quality training and accreditation, ensuring the health of individual people and public health interests. Much of the implementation work to date has been the result of the early mobilisation of academic and not-for-profit organisations, without a centralised coordinating mechanism to facilitate inter-sectoral collaboration, public–private partnerships and rapid capacity increase. Barriers identified in the context of rapid capacity building have included duplication of efforts by groups and capacity limitations. The need for informational continuity of care and the importance of a centralised and well-managed mechanism that allows structures, institutions and care providers to cross-talk has been highlighted (Lionis *et al*., 2018a; vanLoenen *et al*., 2018).

## Discussion

Although several actions have been undertaken by the Greek Ministry of Health and WHO over the past few years, PC in Greece is still far from embracing and utilising to the full extent the key concepts in consensus documents and WHO declarations (IOM, 2001; WHO, 2017; WHO, 2018a). Fragmentation of services and lack of consistent direction in the management of chronic diseases and major public health risks are on-going handicaps (Lionis *et al*., 2017). Poverty, demographic issues, including population ageing, health care expenditures and limited resources and capacities are pressures that NHS will continue to face. Key questions, also, remain regarding the core attitude, knowledge and skill competencies for PC physicians required to serve the Greek population while being confronted with complex biopsychosocial conditions. In this regard, the core aspects of training needed to develop such competences ought to be systematically revised aiming towards an interdisciplinary team approach (Papadakaki *et al*., 2012). The specialty training for GPs has recently expanded from a 4-year cycle to a 5-year cycle to expand baseline skills. However, this is only one of the steps required in terms of how both professionals and the public conceptualise care, PHC, and self-care. For GPs, more comprehensive training is needed to prepare for their role as family physicians in a manner that adequately addresses complex needs of individuals, families and communities. In addition, the Greek NHS still remains conventional, medically focused with a vertical hierarchical structure that promotes health care-seeking behaviours outside the PC level. The long-standing dominance of medically oriented health policy in Greece has impeded efforts to promote a more interdisciplinary integration of care. At the same time, potential for a strong contribution from the nursing profession to PC, through recommended optimum pathways in NCD management, preventive screening, health promotion and home care is still not achieved (Sifaki-Pistolla *et al*., 2017).

The recommendations below are designed to guide current health policy towards an effective integrated PHC model.

(1) Effective human resource planning to increase the number of PC professionals and address existing skill mix imbalances between specialists and GPs and the lack of adequate nursing and allied health professions personnel.
(2) Implementation of a fully operational e-communication, interoperable, system that is sensitive to the pragmatic conditions and accommodates the needs of multidisciplinary teams. Effective use of ICT with a comprehensive medical records system, as reported by Kounalakis *et al*. (2003), should become a high NHS priority.
(3) Orientation of the new PHC units to address major public health issues (i.e., NCD spectrum, including cancer, CVD, diabetes, frailty, traffic accidents) and risk factors (i.e., smoking, obesity, driving behaviour, high consumption of sugar, alcohol, etc.), with interventions encompassing health promotion to change health care-seeking behaviours, prevention, screening and early diagnosis and management of health risks and disease.
(4) Coordinated actions for integrated chronic disease care. The high percentage of people with two or more chronic conditions (i.e., multi-morbid people), necessitates optimal use of available resources and more sophisticated mechanisms to coordinate care. Also, mobilising resources beyond care structures; for example, through participatory initiatives at community level. This, in terms of policy, means substantial investment in ICT and training, with a strong coupling of what such a new approach offers via public awareness campaigns and professional education. A robust action plan could be operationally integrated using the model and tools proposed by Sifaki-Pistolla *et al*. (2017).
(5) Emphasis on integrating public health and PHC, and information flow and exchange in order to inform priorities, opportunities and best practices. Such an effort can be supported by the IPCHS framework on IPCHS (Lionis and Petelos, 2015).
(6) Development of core competencies and implementation of a coordinated continuing education program for PHC professionals. This recommendation addresses the clear need to retrain the PHC practitioners in Greece with a focus on developing the PC team and a culture of interdisciplinary collaboration.
(7) Interprofessional education through a national plan for restructuring PHC training programs, focusing on general practice (changing the structure, curriculum content, teaching and evaluation methods) and other health science disciplines.
(8) Coordination of care by the regional and local health authorities in order to link health care services with other domains and sectors that impact both health promotion and disease prevention. All services should be well tailored with population health care needs and policy planning should consider public perception of PHC.

It must be emphasised that multiple transitions in the context of reform may be difficult to implement successfully, particularly when this takes place rapidly with severely limited resources. Adopting integrated people-centred PHC as the norm (gold standard) throughout settings requires a major change in organisational culture at all levels of the health care system. Several policy papers published by WONCA (2017), including one on improving health globally (vanWeel and Rosser, 2004), could support Greek policy makers towards this action. In addition, PHC policy should showcase contributions to UHC, as commented by vanWeel *et al*., (2018). This investment to PHC should be reflected in training of PHC professionals at community setting, and it is a true challenge for current reform in Greece. The four emerging priorities namely, community-based advocacy to policymakers, collaboration with universities to include PHC as a core component in medical curricula, collaboration with communities to improve public awareness of PHC and engagement with the private sector to focus on PHC and UHC are entirely relevant to the case of Greece.

Lessons learned and recommendations made in this development paper are likely to be relevant to countries where PHC integration is emerging as a key issue in health policy reform. In particular, some of the PHC domains of cross-border relevance are service orientation towards chronic diseases and major public health challenges, integration of public health and advanced training of PHC professionals in multidisciplinary models of care. All of these issues deserve further attention by researchers who will conduct similar analyses in their own countries or settings (Lionis *et al*., 2018b).

## Conclusions

Unravelling Ariadne’s thread shows a clear pathway towards sustainable PHC reform and UHC. Greece is emerging from a prolonged period of financial and refugee crisis during which the austerity measures and the rationing across state provisions have affected the socio-economic status of a large part of the population. At the same time, certain systematic efforts continue to reform PHC, conceptually having a sound base and meeting the tenets of Alma-Ata. However, these efforts necessitate strong political will, immediate and concrete actions, as well as substantial investments, to ensure that rapid transition to the proposed system is successful in ‘leaving no one behind’. Serving as a testament to the resilience of overburdened health care professionals, systems, and, indeed people, this development paper intends to accelerate action for UHC.
